# *CathepsinKCre* mediated deletion o*f βcatenin* results in dramatic loss of bone mass by targeting both osteoclasts and osteoblastic cells

**DOI:** 10.1038/srep36201

**Published:** 2016-11-02

**Authors:** Paula Ruiz, Marta Martin-Millan, M. C. Gonzalez-Martin, Maria Almeida, Jesús González-Macias, Maria A. Ros

**Affiliations:** 1 Instituto de Investigación Marqués de Valdecilla, IDIVAL, Cardenal Herrera Oria s/n. 39011 Santander, Spain; 2 Department of Internal Medicine, HUMV, Hospital Universitario Marqués de Valdecilla, Avenida de Valdecilla s/n, 39008 Santander, Cantabria, Spain; 3 Instituto de Biomedicina y Biotecnología de Cantabria, IBBTEC (CSIC-SODERCAN-Universidad de Cantabria). Albert Einstein 22, 39011 Santander, Spain; 4 Center for Osteoporosis and Metabolic Bone Diseases, University of Arkansas for Medical Sciences and the Central Arkansas Veterans Healthcare System, Little Rock, AR, USA; 5 Departamento de Medicina y Psiquiatría. Facultad de Medicina. Universidad de Cantabria, Cardenal Herrera Oria, s/n. 39011 Santander, Spain.; 6 Red Temática de Investigación Cooperativa en Envejecimiento y Fragilidad (RETICEF), Avenida de Valdecilla, s/n. Santander 39008, Spain.; 7 Departamento de Anatomía y Biología Celular, Facultad de Medicina, Universidad de Cantabria, Cardenal Herrera Oria, s/n. 39011 Santander, Spain.

## Abstract

It is well established that activation of Wnt/βcatenin signaling in the osteoblast lineage leads to an increase in bone mass through a dual mechanism: increased osteoblastogenesis and decreased osteoclastogenesis. However, the effect of this pathway on the osteoclast lineage has been less explored. Here, we aimed to examine the effects of Wnt/βcatenin signaling in mature osteoclasts by generating mice lacking *βcatenin* in *CathepsinK*-expressing cells (*Ctnnb1*^*f/f*^*;CtsKCre* mice). These mice developed a severe low-bone-mass phenotype with onset in the second month and in correlation with an excessive number of osteoclasts, detected by TRAP staining and histomorphometric quantification. We found that WNT3A, through the canonical pathway, promoted osteoclast apoptosis and therefore attenuated the number of M-CSF and RANKL-derived osteoclasts *in vitro*. This reveals a cell-autonomous effect of Wnt/βcatenin signaling in controlling the life span of mature osteoclasts. Furthermore, bone *Opg* expression in *Ctnnb1*^*f/f*^*;CtsKCre* mice was dramatically decreased pointing to an additional external activation of osteoclasts. Accordingly, expression of *CathepsinK* was detected in TRAP-negative cells of the inner periosteal layer also expressing *Col1*. Our results indicate that the bone phenotype of *Ctnnb1*^*f/f*^*;CtsKCre* animals combines a cell-autonomous effect in the mature osteoclast with indirect effects due to the additional targeting of osteoblastic cells.

Evidence accumulated during the last few years has established that the Wnt/βcatenin pathway is critical for bone formation and skeletal homeostasis^1^,^2^. The initial evidence came from the identification of loss-of-function (LOF) and gain-of-function (GOF) mutations in the human *LRP5* gene (low-density-lipoprotein receptor-related protein 5; a co-receptor of the Wnt/βcatenin pathway) as responsible for the Osteoporosis-Pseudoglioma Syndrome and for the hereditary High Bone Mass trait, respectively^3^,^4^. The bone phenotypes of these mutations were later reproduced in genetically modified mouse models of *Lrp5* function, further highlighting the anabolic role of Wnt/βcatenin signaling in bone^5^.

Genetic studies in mice manipulating *βcatenin*, the obligatory component of canonical Wnt signaling, have established that βcatenin in the osteoblast lineage increases bone mass through different mechanisms, depending on the specific differentiation stage of the cell^6^,^7^,^8^,^9^,^10^,^11^. For instance, activation of canonical Wnt signaling by stabilization of βcatenin in mesenchymal progenitor cells, plays an essential role in controlling osteoblast versus chondrocyte differentiation and in promoting osteoblast proliferation and differentiation^6^,^7^,^9^. Moreover, Wnt/βcatenin signaling in osteoblast and osteocytes upregulates the osteoclasts inhibitor factor osteoprotegerin (OPG) and thereby inhibits osteoclast formation and bone resorption^10^,^11^. Bone loss mediated by decreasing OPG expression is also observed in the conditional deletion of *βcatenin* in *type II Collagen* expressing cells^12^. Thus, a cell- autonomous mechanism promoting proliferation and maturation of osteoblasts together with an indirect non cell-autonomous mechanism, mediated by an OPG-dependent reduction in osteoclast numbers, contribute to the high bone mass phenotype.

The involvement of osteoclasts as mediators of some of the effects of Wnt/βcatenin signaling in the osteoblast lineage, the ubiquity of WNT proteins and the expression of components of the Wnt canonical pathway in the osteoclast lineage raised the question of whether this pathway could also play a cell-autonomous role in the osteoclast biology^13^,^14^. To explore this possibility, several attempts have been made to genetically manipulate *βcatenin* in the osteoclast lineage^15^,^16^,^17^,^18^. For example, the *LysozimeMCre (LysMCre*) deleter line, which drives recombination specifically in monocyte/macrophages and neutrophils^19^ has been used to perform gain^17^ and loss^15^,^16^,^17^ of function of *βcatenin* in osteoclast precursors. *βcatenin* deletion in *LysozimeM* expressing cells leads to osteopenia by increasing osteoclastogenesis but without altering OPG levels. These results suggest that direct actions of Wnt/βcatenin signaling in the osteoclast lineage contribute to bone homeostasis^15^,^16^,^17^. However, in these models the genetic manipulation of *βcatenin* is not restricted to the osteoclasts but it also affects other cell types such as macrophages and neutrophils which could potentially contribute to the phenotype. In the present work, we aimed to study the function of Wnt/βcatenin specifically in mature osteoclasts using the *CtsKCre* deleter line, which is commonly used to target osteoclasts at their late stage of differentiation^20^,^21^,^22^. *CtskCre;βcatenin* mice developed severe low-bone-mass caused by excessive osteoclast numbers. *In vitro* experiments showed that Wnt signaling decreased osteoclast numbers by reducing their life span in a cell-autonomous manner. We also identified a population of osteogenic cells located in the inner layer of the periosteum that express *Ctsk* postnataly. The *CtskCre* mediated removal of *βcatenin* in this population of osteoblasts, most probably, led to reduction in OPG production, thereby, contributing to the excessive osteoclast formation and dramatic loss of bone mass.

## Results

### Generation of mice with specific *βcatenin* deletion in *Ctsk*-expressing cells

**Figure 1:**
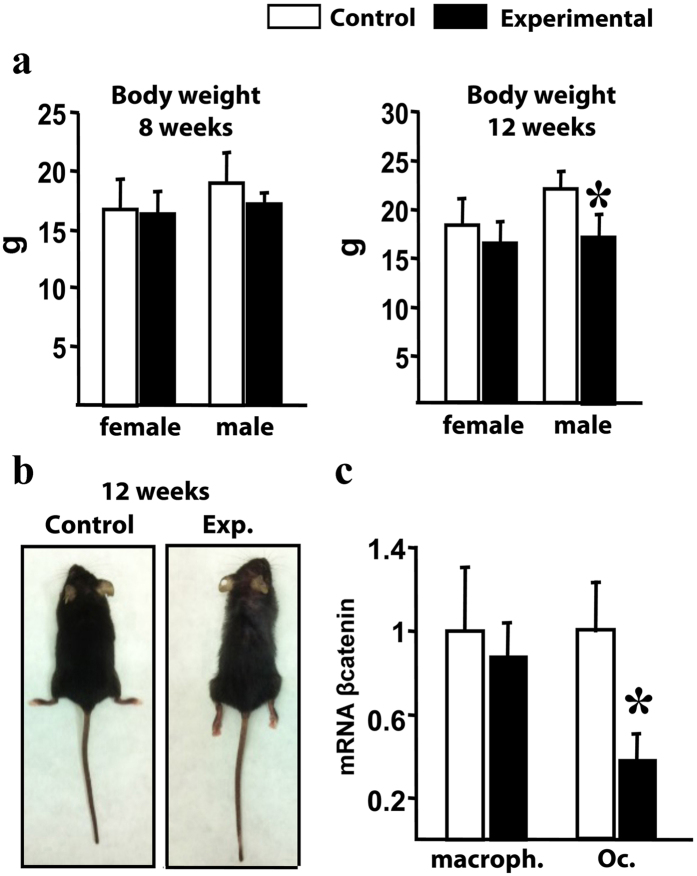
External appearance of *Ctnnb1*^*f*/*f*^*;CtsKCre* mice and *βcatenin* expression in macrophages and osteoclasts. (**a**) Comparison of body weight between control and experimental littermate mice at 8 and 12 weeks of postnatal life (g, grams). (**b**) External body appearance of 12-week-old control and experimental mice. (**c**) RT-PCR quantification of *βcatenin* transcripts in macrophages and osteoclasts developed from non-adherent bone marrow cells cultured in the presence of M-CSF for 4 days, and M-CSF plus RANKL for 5 days, respectively (n = 4). *Bars, Mean* ± *SD;* *P < *0*.*05*.

Conditional inactivation of *βcatenin* (MGI: *Ctnnb1*) in mature osteoclasts was performed using the *βcatenin* floxed allele (*Ctnnb1*^*f*^;^23^) and the *CathepsinK;Cre* line (*CtsKCre*;^21^) that harbors the *Cre recombinase* at the *CathepsinK* locus. We generated two cohorts (males and females) of mice lacking *βcatenin* in *CtsK*-expressing cells, hereafter referred to as *Ctnnb1*^*f/f*^*;CtsKCre* or experimental mice (n = 13 males and 17 females), and they were compared with the corresponding *Ctnnb1*^*f/f*^ littermate animals, considered as controls (n = 21 males and 20 females). *Ctnnb1*^*f/f*^*;CtsKCre* mice were born at the expected Mendelian ratio and their gross external aspect and size were indistinguishable from control mice. However, during the second month of postnatal life, the experimental *Ctnnb1*^*f/f*^*;CtsKCre* mice became clearly distinct from their control littermates due to an abnormal gait, malposition of the hindlimbs and sparse fur appearance as shown for 12-weeks old animals in Fig. 1b. Differences in body weight were also seen in males at this age (Fig. 1a). All experimental animals died between 14 and 15 weeks of age, a time when animals appeared sick.

Excision of the *βcatenin* gene was assessed by qRT-PCR in *ex vivo* cultures of bone marrow (BM)-derived macrophages and osteoclasts obtained from the femur of experimental and control 4–6 week-old mice. *βcatenin* mRNA levels in macrophages from *Ctnnb1*^*f/f*^*;CtsKCre* mice were indistinguishable from controls while in osteoclasts *βcatenin* levels were reduced by approximately 60% (Fig. 1c). The magnitude of the reduction in *βcatenin* was similar to the one seen with other Cre-deleter models^10^,^11^. The remaining *βcatenin* seen in cultures from experimental mice was, most probably, due to the presence of non-*CtsK*-expressing cells, of the hematopoietic or mesenchymal lineages.

### *βcatenin* deletion in *CtsK*-expressing cells causes a severe loss of bone mass

**Figure 2:**
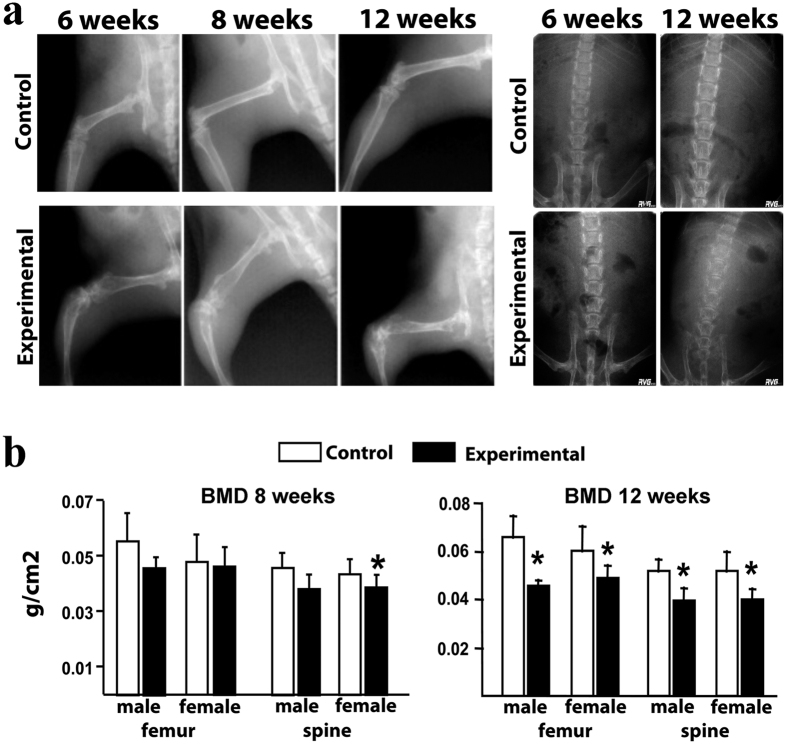
X-ray and BMD analysis of *Ctnnb1*^*f/f*^*;CtsKCre* mice. (**a**) Radiographic imaging of the hindlimb and spine of experimental and control littermates at the stages indicated on the top. (**b**) Bone mineral density (BMD) (bone mineral content/total volume) of the femur and spine of control and experimental littermates at 8 and 12 weeks of postnatal life. *Bars, Mean* ± *SD;* *P < *0*.*05*.

The hindlimb malposition and abnormal gait of *Ctnnb1*^*f/f*^*;CtsKCre* mice pointed to a skeletal phenotype. To start characterizing this phenotype we performed a sequential X-ray examination of the hindlimbs and spine of both experimental and control animals. As early as 6 weeks of age, experimental animals exhibited cortical thinning and alterations in shape including widening of the distal third of the femur and proximal third of the tibia (Fig. 2a, left panels). Furthermore, the secondary ossification center in the distal femur and proximal tibia were markedly misshapen in experimental animals. Poor mineralization of the vertebral bodies was also evident at 12 weeks of age (Fig. 2a, right panels). In line with the X-ray analysis, monthly DEXA bone mineral density BMD analysis showed a reduction of bone mass in the spine of *Ctnnb1*^*f/f*^*;CtsKCre* females, in the second analysis performed at 8 weeks and in both the spine and femur of females and males at 12 weeks (Fig. 2b).

**Figure 3:**
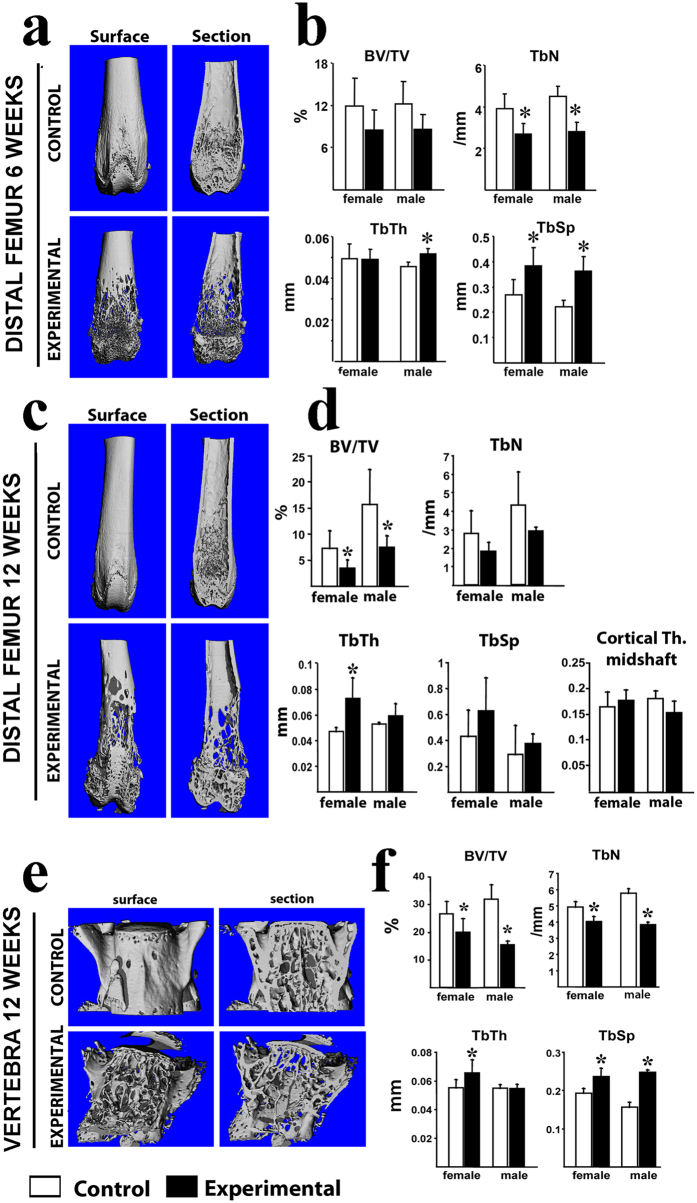
Micro–computed tomography analysis of *Ctnnb1*^*f/f*^*;CtsKCre* mice showing a severe loss of bone mass. (**a**) Representative μCT images of the distal femur of the surface and mid-section of 6-week-old control and experimental littermates. (**b**) Histomorphometric quantification showing changes in trabecular number, trabecular space and trabecular thickness. (**c**) Representative μCT images of the distal femur of the surface and mid-section of 12-week-old control and experimental littermates. (**d**) Histomorphometric quantification showing changes in bone volume, and trabecular thickness. BV/TV: bone volume fraction; Tb.N: trabecular number; Tb.Th: trabecular thickness; Tb.Sp: trabecular separation. Ct.Th: average cortical thickness. (**e**) Representative μCT images of L5 vertebra of 12-week-old control and experimental littermates. (**f**) Histomorphometric quantification showing changes in experimental mice in all bone parameters analyzed. Surface and mid-sections are shown (indicated on the top). BV/TV: bone volume fraction; TbN: trabecular number; TbTh: trabecular thickness; TbSp: trabecular separation. CtTh: average cortical thickness. *Bars, Mean* ± *SD;* *P < *0*.*05*

To examine the bone microarchitecture in *Ctnnb1*^*f/f*^*;CtsKCre* mice, we performed micro-computed tomography (μCT) of the femur and fifth lumbar vertebra (L5) of 6 and 12 week-old animals. The μCT images showed a clear bone loss in the distal femur in 6 week-old animals (Fig. 3a). Compared with respective controls, experimental female and male mice had decreased trabecular number (TbN) and increased trabecular separation (TbSp) (Fig. 3b). However, the bone volume fraction (BV/TV) was not significantly different from controls (Fig. 3b). At 12 weeks of age, μCT scans of the distal femur revealed a dramatic loss of both cortical and trabecular bone (Fig. 3c) and both female and male experimental animals showed 50% decrease in BV/TV (Fig. 3d). However, no significant changes were detected in trabecular number and spacing but trabecular thickness was significantly increased in females (Fig. 3d). Moreover, despite the enormous damage seen in cortical bone at the distal end, the average cortical thickness (Th.) at the midshaft was unaffected in both male and female *Ctnnb1*^*f/f*^*;CtsKCre* mice (Fig. 3d).

Like in the femur, cortical and trabecular bone were dramatically reduced in vertebra (L5) of 12-week-old experimental mice (Fig. 3e). Specifically, female and male mice exhibited a decrease in BV/TV, trabecular number, and an increase in trabecular spacing (Fig. 3f). Trabecular thickness was significantly higher in the experimental females (Fig. 3f).

Together, these results indicate that *βcatenin* deletion in *CtsK*-expressing cells causes a severe loss of both cortical and trabecular bone that started after the first month of postnatal life and worsen progressively.

### *Ctnnb1* ^*f/f*^ *;CtsKCre* mice exhibit normal bone development up to 4 weeks of age

**Figure 4:**
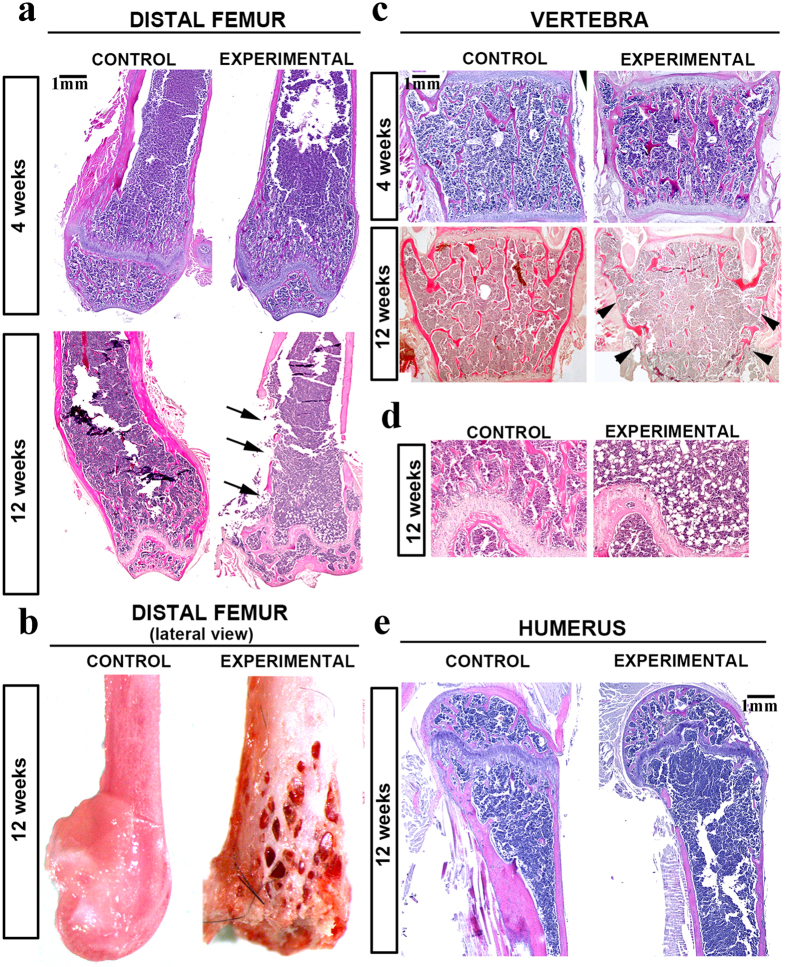
Temporal histological analysis showing the late onset of the phenotype. (**a**) Sequential analysis of the distal femur shows normal bone development in experimental mice up to 4 weeks of postnatal life while wide areas of cortical bone loss (arrows) are observed in 12-week-old experimental animals. (**b**) Pictures of fresh dissected distal femur showing the dramatic bone loss of experimental animals. (**c**) The sequential analysis of lumbar vertebrae shows normal bone development in experimental mice up to 4 weeks with a dramatic bone loss phenotype observed in 12-week-old experimental animals. (**d**) Detail of the bone marrow of the 12-week-old femurs in (A) showing the extensive adiposity characteristic of experimental animals. (**e**) Hematoxylin-eosin stained histological sections of the proximal humerus of control and experimental. Scale bar, 1000 μm.

A sequential Hematoxylin-eosin examination of femur and vertebra sections was performed to further investigate the onset of the phenotype and the bone structure of experimental animals. Up to 4 weeks of postnatal development, both femur and vertebra appeared normal in experimental animals (Fig. 4a,c). However, a dramatic reduction in both the trabecular and cortical bone compartments was evident by 12 weeks old *Ctnnb1*^*f/f*^*;CtsKCre* mice (Fig. 4a–c) with cortical bone absent from wider areas of the distal femur (arrows in Fig. 4a) and vertebra (arrowheads in Fig. 4c) in 12 weeks old experimental animals. The loss of bone in distal femur was obvious in the fresh bone (Fig. 4b) and accompanied by extensive bone marrow adiposity (Fig. 4d). It should be noted, that the long bone phenotype was not restricted to the distal femur, as similar findings were observed in the proximal tibia (not shown) and humerus (Fig. 4e).

The results of the histological analysis confirm that Wnt/βcatenin signaling in *CtsK-*expressing cells is not necessary for normal skeletogenesis up to the end of the first month of postnatal life and suggest that excess bone destruction, starting between 4 and 6 weeks, might be the primary mechanism involved in the bone loss phenotype.

### *Ctnnb1* ^*f/f*^ *;CtsKCre* mice exhibit increased osteoclast numbers

**Figure 5:**
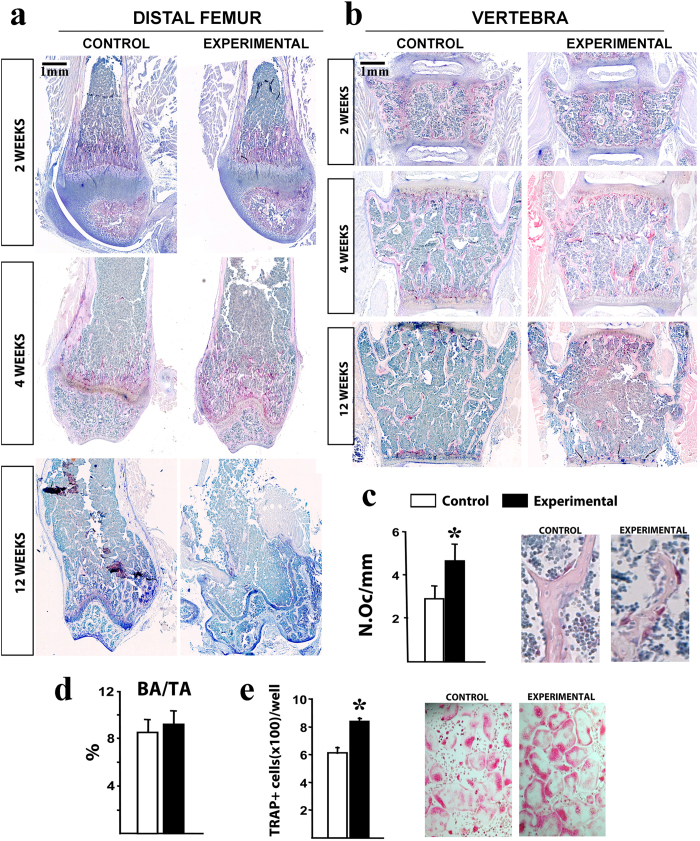
Increased osteoclast numbers in experimental animals. (**a**,**b**) TRAP staining show excess osteoclasts (stained in red) in 4-week-old experimental animals. (**c**) Osteoclast numbers per mm bone surface in cancellous bone of L2-4 vertebra of 4-week-old control (n = 6) and experimental (n = 6) littermates and representative histological photomicrographs of vertebral trabecular bone. (**d**) Histomorphometric analysis of longitudinal decalcified sections of L2–L4 vertebrae from 4-week-old female mice (n = 6 mice per group) showing no difference in bone area per tissue area (BA/TA) between control and experimental animals. (**e**) Quantification of TRAP-positive cells generated from bone marrow cells cultured with M-CSF and RANKL for 5 days and representative microphographs of the culture dishes. *Bars, Mean* ± *SD;* *P < *0*.*05*. Scale bar, 1000 μm.

To determine whether altered bone resorption was the cause of the bone phenotype in *Ctnnb1*^*f/f*^*;CtsKCre* mice, osteoclast numbers were evaluated in histological sections of femur and vertebra. Tartrate-resistant acid phosphatase (TRAP) specific staining revealed an increase in osteoclasts in the distal femur and lumbar vertebra of 4-week-old experimental mice as compared to littermate controls (Fig. 5a,b). Indeed, quantitation of osteoclasts in representative vertebral sections confirmed that osteoclast numbers were significantly increased in experimental mice (Fig. 5c) at 4 weeks of age just before the phenotype was apparent. However, and in line with the μCT results, the bone area per total area did not show differences between control and experimental mice (Fig. 5d), confirming the late onset of the osteopenic phenotype. Osteoclasts were barely detectable in 12-week-old experimental animals, likely because a great portion of the cancellous bone was already lost (Fig. 5a,b). Overall, our results strongly suggest that an increase in osteoclast numbers is the primary cause of the bone defects in *Ctnnb1*^*f/f*^*;CtsKCre* mice.

### Wnt/βcatenin signaling promotes osteoclast apoptosis and attenuates osteoclast numbers *in vitro*

We also assessed the number of osteoclast progenitors in the BM by culturing BM-cells obtained from the femur of control and experimental mice in the presence of RANKL and M-CSF for 5 days. The number of osteoclasts developed in cultures from *Ctnnb1*^*f/f*^*;CtsKCre* mice was significantly higher than in cultures from littermate controls (Fig. 5e). It should be noted that *βcatenin* deficient osteoclasts exhibited normal morphology indicating that *βcatenin* is dispensable for the intracellular cytoskeleton architecture and cell-cell adhesion of osteoclasts (Fig. 5e). As suggested in previous studies, plakoglobin may substitute for βcatenin in these functions^6^,^24^,^25^.

**Figure 6:**
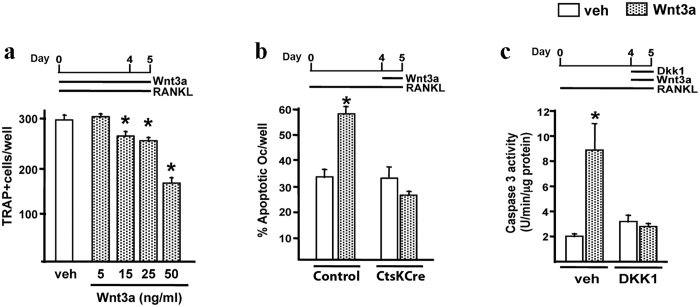
WNT3A induces osteoclast apoptosis via the canonical Wnt signaling pathway. (**a**) Number of TRAP-positive cells generated from non-adherent bone marrow cells from C57BL/6 control mice cultured with M-CSF, RANKL and either vehicle (veh) or different doses of WNT3A recombinant protein as indicated. (**b**) TUNEL assay in mature osteoclasts generated from bone marrow cells from *Ctnnb1*^*f/f*^*;CtsKCre* mice and their respective littermate controls, cultured with M-CSF and RANKL for 5 d and treated with either veh or WNT3A (50 ng/ml) for 24 h. (**c**) Caspase 3 activity, expressed in arbitrary fluorescent units, in mature osteoclasts generated from non-adherent bone marrow cells from wild-type mice, and treated with veh, WNT3A (50 ng/ml) or DKK1 (1 μg/ml) for 24 h. *Bars, Mean* ± *SD;* *P < *0*.*05*.

In accordance with this observation, addition of recombinant WNT3A dose-dependently decreased the number of osteoclasts derived from BM of wild type mice in the presence of M-CSF and RANKL (Fig. 6a). Thus, stimulation of the canonical Wnt signaling attenuates the number of osteoclasts derived from BM cells *in vitro*. Because the genetic manipulation in our experimental mice occurs in a late phase of osteoclast differentiation, most likely when osteoclast are already postmitotic, we considered the alternative possibility of Wnt signaling modifying their life span.

Osteoclasts have a short life span and die by apoptosis, therefore, changes in their life span may alter osteoclast numbers. We next examined the role of Wnt/βcatenin signaling in osteoclasts apoptosis. Addition of WNT3A to osteoclast cultures from BM of control littermates mice stimulated osteoclast apoptosis, as determined by the TUNEL assay (Fig. 6b). The pro-apoptotic effect of WNT3A was abrogated in osteoclast cultures obtained from *Ctnnb1*^*f/f*^*;CtsKCre* mice (Fig. 6b), confirming βcatenin requirement for this effect. Addition of DKK1 to the cultures also blunted the pro-apoptotic actions of WNT3A in osteoclasts as determined by caspase 3 activity (Fig. 6c). These results show that canonical Wnt/βcatenin signaling promotes osteoclast apoptosis *in vitro*.

### A decline in OPG in cells other than osteoclasts contributes to the low-bone-mass in *Ctnnb1* ^*f/f*^; *CtsKCre* mice

**Figure 7:**
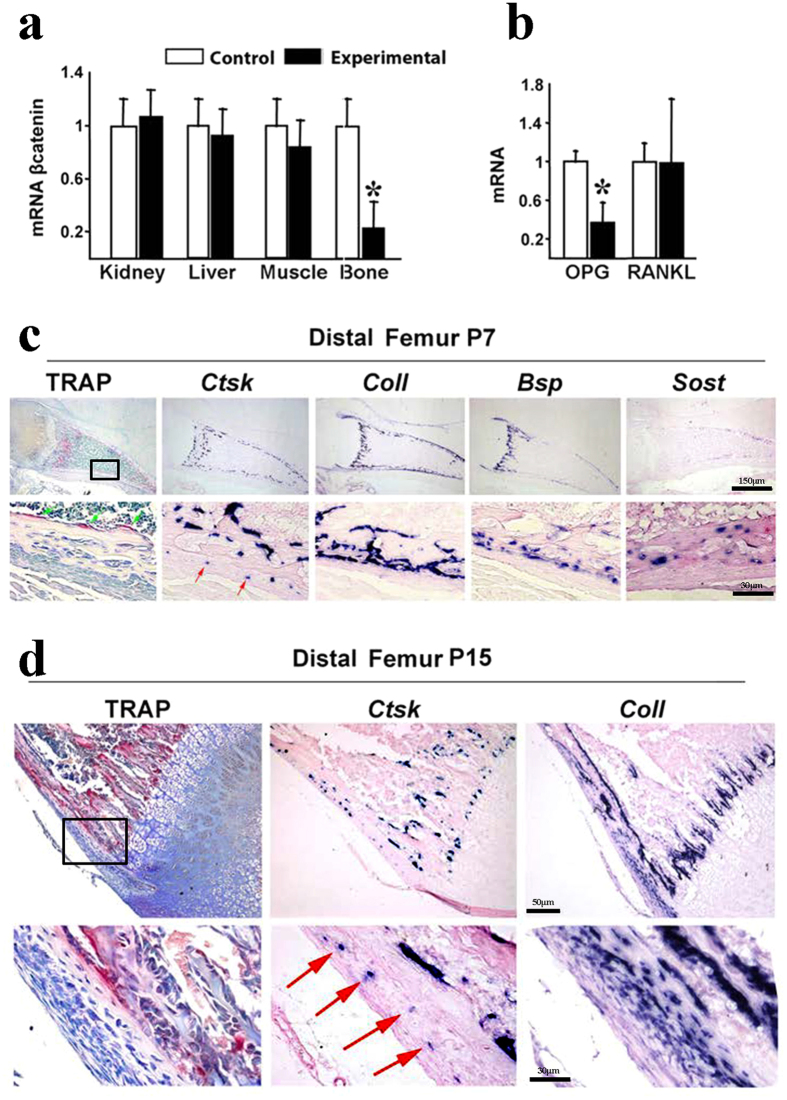
Reduced *βcatenin* and *Opg* mRNA expression in experimental bone tissue and pattern of *CtsK* expression in bone. (**a**) Quantitative RT-PCR analysis of *βcatenin* mRNA from a battery of tissues harvested from 4-week-old mice (n = 3) showing specific but disproportionate reduction in bone. (**b**) Quantitative RT-PCR of *Opg* and *Rankl* mRNA in tibia of control mice and their experimental littermates (n = 4). (**c**) TRAP and *in situ* hybridization for *CtsK, Col1, Bsp* and *Sost* in consecutive sections of the distal femur of a P7 wild type animal. The marked square in the top row (bar: 150 μm) is amplified in the bottom row (bar: 30 μm). Note the presence of *CtsK* expressing cells (red arrows) in the inner layer of the periosteum that however are negative for TRAP staining and other osteoblastic markers. (**d**) TRAP and *in situ* hybridization for *CtsK* and *Col1* in consecutive sections of the distal femur of a P15 wild type animal. The marked square in the top row (bar: 50 μm) is amplified in the bottom row (bar: 30 μm). At this stage the *CtsK* positive cells of the cambium layer also express *Col1. Bars, Mean* ± *SD;* *P < *0*.*05*.

The low-bone-mass phenotype in *Ctnnb1*^*f/f*^*;CtsKCre* mice is much more severe than that reported for *Ctnnb1*^*f/f*^*/LysMCre* mice^15^,^16^,^17^. Since the absence of *βcatenin* in osteoclasts is common to both models, the discrepancy in the phenotype suggests the involvement of additional cell targets in one or both models. Indeed, several observations pointed to additional contribution of non-osteoclast cells to the phenotype of *Ctnnb1*^*f/f*^*;CtsKCre* mice. First, the similarity of the bone phenotype with that of mice in which *βcatenin* was removed from osteoblasts and osteocytes, or chondrocytes suggested a common causal mechanisms^11^,^12^,^24^,^26^. Second, the evaluation of *βcatenin* mRNA expression level in the experimental bone (assessed in tibia from 4-week-old animals) was reduced by 70%, much more than expected if only osteoclasts, which represent only a scanty 3–5%, proportion of bone cells, were responsible for this decrease (Fig. 7a).

Since a decline in OPG is the main culprit for the increased resorption in mice in which *βcatenin* was deleted from osteoblast but not from osteoclast lineage cells, we measured *Opg* mRNA expression in the whole tibia in control and *Ctnnb1*^*f/f*^*;CtsKCre* mice. *Opg* mRNA levels were significantly decreased in experimental mice (Fig. 7b). In contrast, RANKL expression was indistinguishable from littermate controls (Fig. 7b). The decrease in OPG in *Ctnnb1*^*f/f*^*;CtsKCre* mutant mice provides an additional cause for the increase in osteoclast numbers and strongly suggests that *CtsK*-Cre targets cells other than osteoclasts.

To identify this putative cell type we analyzed the mRNA expression pattern of *CtsK* by *in situ* hybridization in tissue sections of femur and vertebra from embryonic day (E) 13.5 to 4 weeks of postnatal development (Supplemental Fig. 1 and not shown). *CtsK* expression was first detected at E16.5 and, during this and subsequent developmental stages, the pattern of *CtsK*-expressing cells coincided with the distribution of TRAP positive cells, as expected (Fig. 7c and Supplemental Fig. 1).

Importantly, the expression of *CtsK* was also found in TRAP negative cells located in the inner layer of the periosteum (red arrows in Fig. 7c). The periosteum is a thick sheet that covers the outer surface of bones and is composed of two distinct layers: an outer fibrous layer that lends structural integrity and an inner layer that has osteogenic potential. The fibrous layer is primarily composed of fibroblasts, collagen, and elastin while the inner layer, also called the cambium layer, is highly cellular and includes differentiated osteogenic progenitors, adult mesenchymal progenitor cells and osteoblasts^27^,^28^,^29^.

The periosteal *CtsK*-expressing cells appeared scattered in the cambium layer, not in direct contact with the bone surface, but adjacent to the line of cuboid osteoblasts (Fig. 7c,d). At postnatal (P) day 7, these cells were negative for *Collagen type1 (Col1*), *Bone sialoprotein (Bsp*), and *sclerostin (Sost*) (Fig. 7c). Most interestingly, at P15, these cells also expressed *Col1* but not *Bsp* or *Sost*, suggesting that they are a subpopulation of undifferentiated osteogenic cells (Fig. 7d). It should be noted that we could not detect *CtsK* expression in osteocytes or in the Ranvier groove at this stage (Fig. 7). Together, these results strongly indicate that the *CtsKCre* line targets cells other than osteoclasts in bone and that deletion of *βcatenin* in these cells contributes to the loss of bone mass in *Ctnnb1*^*f/f*^*;CtsKCre* mice.

## Discussion

The aim of this work was to examine the function of Wnt/βcatenin signaling specifically in mature osteoclasts. Surprisingly, the removal of *βcatenin* at a late stage of osteoclast differentiation using the *CtsKCre* line caused a much more severe low-bone-mass phenotype than when *βcatenin* is removed from early myeloid precursors using the *LysMCre* line^15^,^16^,^17^. While *Ctnnb1*^*f/f*^*;LysMCre* mice show a mild reduction in the femur trabecular bone^15^, the *Ctnnb1*^*f/f*^*;Ctsk-cre* mutants show a dramatic loss of both trabecular and cortical bone in both long bones and vertebra. Because in both animal models the mature osteoclast is deficient in *βcatenin*, it is reasonable to assume that the discrepancies in the phenotype rely on additional targets, distinct from osteoclasts, present in one or both of these models. Also, it should be considered that *Ctnnb1*^*f/f*^*;CtskCre* mice lack a functional allele of *Ctsk*, one of the most potent collagenases^30^. This raises the question of whether the degradation of ECM proteins, such as collagen and elastin, could be impaired in our experimental mice and contribute to the phenotype. However, this possibility can be discarded because *Ctsk* heterozygous mutant have no bone phenotype^31^,^32^ and because the *Ctnnb1*^*f/f*^*;CtskCre* phenotype is characterized by excessive rather than limited osteoclasts function.

Here, we have focused on the causes of the severe low-bone-mass in *Ctnnb1*^*f/f*^*;CtsKCre* mice and show that it associates with excessive osteoclast numbers evident at the end of the first month of postnatal life and coincident with the onset of the phenotype. The increase in osteoclast numbers results from the combination of two mechanisms. First, the removal of *βcatenin* in mature osteoclasts extends their life span in a cell-autonomous manner and second, the additional activity of the *CtsKCre* line in a sub-population of osteoblasts leads to increased osteoclastogenesis through the reduction in OPG production.

An essential role for *βcatenin* in controlling osteoclastogenesis has been previously demonstrated. It has been shown that WNT3A reduces osteoclast numbers by sustaining M-CSF–induced proliferation of osteoclasts precursors and preventing their differentiation into mature osteoclasts^15^,^17^. Recently, it has been shown that WNT3A suppresses RANKL-mediated NFATc1 expression in osteoclast precursors, while activates the cAMP/PKA pathway, as mechanisms by which WNT3A blunts osteoclast differentiation^18^.

Our *in vitro* experiments provide compelling evidence for a role of cell-autonomous Wnt/*βcatenin* signaling in reducing the life span of osteoclasts. The pro-apoptotic effect of Wnt/βcatenin in osteoclasts may occur all along the osteoclast differentiation life as an additional mechanism together with the attenuation of differentiation, and our experiments suggest that this effect is still present at the end of the osteoclastic differentiation process. Several studies have demonstrated that modulation of osteoclasts life span leads to changes in bone mass^33^. For example, estrogens protect the skeleton, in part, via pro-apoptotic effects in osteoclasts^21^,^34^. Indeed, deletion of *ERα* in the osteoclast lineage cells, similar to deletion of *βcatenin*, increases osteoclast numbers and decreases bone mass. In contrast to estrogens, glucocorticoids cause loss of bone mass, at least in part, via prolongation of osteoclast life span. Interestingly, and similar to estrogens and glucocorticoids, Wnt/βcatenin signaling exerts an opposite effect on the life span of osteoblasts and osteoclasts i.e. it increases the life span of osteoblasts and decreases the life span of osteoclasts^35^. Similar to our findings in osteoclasts, WNT3A/βcatenin mediate the effects of hypoxia reoxygenation on rat cardiomyoblasts cell death^36^. Moreover, Wnt/βcatenin signaling decreases melanoma cell invasiveness by a mechanism that enhances the expression of pro-apoptotic proteins^37^.

Several findings in *Ctnnb1*^*f/f*^*;CtsKCre* mice pointed to the involvement of cells other than osteoclasts in the bone phenotype of these mice. First, the reduction in the expression of *βcatenin* in bone is much higher than expected from the sole removal in osteoclasts, a minority cell type in bone. Second, the expression of *Opg*, a well-known target of *βcatenin* in osteoblasts, is dramatically reduced in bone. Third, the bone phenotype, including the excessive bone marrow adipocytes, is reminiscent of the phenotype exhibited by mice in which *βcatenin* is deleted in osteoblasts, osteocytes or chondrocytes^11^,^12^,^24^,^26^,^38^. These observations, together with the identification in the inner periosteal layer of cells that are TRAP-negative but concomitantly express *CtsK* and *Col1*, support the conclusion that a sub-population of osteogenic cells contributes to the phenotype via indirect control of osteoclastogenesis. Because the *CtsK*-expressing cells identified here are located in the inner layer of the periosteum and express *Col1* at P15, we propose that they are a sub-population of osteoblastic cells. Although it has been reported that osteocytes can express *CtsK* under certain circumstances^39^, our study failed to detect cells that concomitantly expressed *Sost* and *CtsK* during the period studied.

Besides Wnt/βcatenin, Bmp signaling is another important pathway regulating both bone formation and bone resorption and it is known that both pathways interact in complex and varied ways^2^,^40^. Thus, Bmp signaling, through the Bmpr1a, positively regulates sclerostin expression as well as canonical Wnt signaling^41^,^42^. In addition, Wnt/βcatenin signaling is required for the Bmp osteogenic effects^43^ and Bmp2 directly enhances osteoclastic differentiation and survival by regulating expression of RANKL and CSF1^44^,^45^. While less is known on Wnt/βcatenin modulation of Bmp signaling in bone, recent studies have shown upregulation of Bmp ligands by Wnt/βcatenin signaling^46^. Thus, it is possible that Bmp signaling is altered in *Ctnnb1*^*f/f*^*;CtsKCre* mice, an issue that certainly deserves further analysis.

Interestingly, *CtsK* expression has also been recently described surrounding the physis, in the anatomical structure called the groove of Ranvier, which contains osteochondro-progenitor cells that contribute to bone development^47^. However, despite careful analysis we could not detect *CtsK* expressing cells in such location, ruling out the participation of this structure in the phenotype of our experimental mice.

## Methods

### Mutant mice

The *βcatenin* floxed allele (*Ctnnb1*^*f*^)^23^ was obtained from Jackson Laboratory (Bar Harbor, Maine) and the *CtsKcre* line^21^ was provided by Dr. Shigeaki Kato (Institute of Molecular and Cellular Biosciences, University of Tokyo). Mice were genotyped by PCR following standard procedures. All animal procedures were conducted accordingly to the EU regulations and 3R principles and reviewed and approved by the Bioethics Committee of the University of Cantabria. A total of 13 males and 17 females of experimental mice and 21 males and 20 females for control animals were used.

### DEXA bone mineral density

Bone mineral density (BMD) measurements were performed in 10 animals per genotype by DEXA using a PIXImus-II densitometer (GE Healthcare Lunar, Piscataway, NJ). Total spine and right femur BMD measurements were performed according to the manufacturer.

### Micro-computer tomography (μCT)

μCT analysis of the fifth lumbar vertebra and right femur of 10 mice per genotype was performed into 12.3 mm diameter scanning tubes and imaged (μCT40, Scanco Medical, Basserdorf, Switzerland). Scans were integrated into 3-D voxel images (1024 × 1024 pixel matrices for each individual planar stack) and a Gaussian filter (sigma = 0.8, support = 1) was used to reduce signal noise. A threshold of 200 was applied to all analyzed scans. Scans were done at medium resolution (E = 55 kVp, I = 145 μA, integration time = 200ms). The entire vertebral body was scanned with a transverse orientation excluding the pedicles and articular processes. Manual analysis excluded the cortical bone and the primary spongiosa from the analysis. All trabecular measurements were made by manually drawing contours every 10 to 20 slices and using voxel counting for bone volume per tissue volume and sphere filling distance transformation indices without assumptions about the bone shape as a rod or plate for trabecular microarchitecture. Cortical thickness was measured at the femur mid-diaphysis. Densitometry, and μCT analyses were performed blind.

### Bone histology

Three lumbar vertebrae (L2-4) and left femur of 6 animals for each genotype, were fixed in 4% paraformaldehyde overnight at 4 °C and decalcified in 9% EDTA (pH7.4) for a variable time period, depending on the stage, before embedding in paraffin. For the histomorphometric analysis, osteoclasts were stained for tartrate-resistant acid phosphatase (TRAP) and measurements performed with Fiji software. Data are reported using the nomenclature recommended by the American Society for Bone and Mineral Research.

### Cell culture

To quantify osteoclast progenitor cells, bone marrow (BM) was flushed out from long bones of 3 mice for each genotype and plated in triplicates at a density of 50,000 cells/cm^2^ in 48 well plates. After 4–5 days of culture in α-MEM medium (Invitrogen), supplemented with 10% FBS, 1% PSG, 30 ng/ml M-CSF, and 30 ng/ml sRANKL (R&D Systems) the cells in the plate were fixed with 10% neutral buffered formaldehyde for 15 min and osteoclasts detected by staining for TRAP. Non adherent BM cells depleted of stromal cells were used for the rest of culture experiments.

### Quantitative real-time PCR (qRT-PCR)

Total RNA of 4 animals per genotype, was extracted with TRIzol reagents (Life Tecnologies). 1μg total RNA was used to produce first-strand cDNA using the m-MLV RT enzyme (Invitrogen). qRT-PCR was carried out in duplicates using the PreMix Ex taq (Takara) and the data were analyzed using the Biorad software. The primers and probes for *βcatenin* [Mm01350385_g1 (fam)], and gapdh [Mm99999915_g1 (vic)] were manufactured by the TaqMan Gene. Expression Assays service (Applied Biosystems).

RT-PCR for *Rankl* and *Opg* expression was analyzed in a termocycler (Step One Plus Real Time PCR Systems, Applied Biosystems) using the SYBRGreen PCR Master Mix (Invitrogen). The primers used were: *Rankl* (F: 5′-AAATTAGCATTCAGGTGTCC-3′ and R: 5′-AATGTTCCACGAAATGAGTC-3′) and *Opg* (F: 5′-AAAAATGTCCAGATGGGTTC-3′ and R: 5′-ACACAGGGTGACATCTATTC-3′). The relative expression of mRNA obtained was normalized to tubulin, used as housekeeping (F: 5′-GCAGTGCGGCAACCAGAT-3′ and R: 5′-AGTGGGATCAATGCATGCT-3′). Relative mRNA expression levels were normalized to the mentioned housekeeping using the ΔCt method^48^.

### Apoptosis assays

Osteoclasts derived from purified non-adherent bone marrow osteoclast precursors isolated from 3 animals for each genotype were treated with WNT3A once osteoclasts had developed in the culture plate in triplicates. After 24 hours of treatment, the cultures were fixed and subject to TUNEL and TRAP staining. The total number of osteoclasts and the number of apoptotic osteoclasts in the plate were quantified. Osteoclasts were considered apoptotic when at least one of their nuclei was TUNEL positive. The TUNEL method was performed using the FragEL DNA fragmentation detection kit (EMD Chemicals, San Diego, CA) before staining for TRAP. Multinuclear TRAP-positive and TUNEL-positive cells were enumerated. Caspase-3 activity was measured by determining the degradation of the fluorometric substrate DEVD (Biomol Research Laboratories, Plymouth Meeting, PA), and protein concentration was measured using a Bio-Rad detergent-compatible kit (Bio-Rad Laboratories, Hercules, CA).

### *In Situ* Hybridization

*In situ* hybridization was performed in sections using digoxigenin-labeled antisense riboprobes. The probes used were *Col1, Bsp* and *Sost*^8^. A specific antisense RNA probe for *CtsK* was generated using the following primers: CtsK-F: gaccactgccttccaatacg and CtsK-R:ctgctggaggactccaatgt. A minimum of three mice of each stage were analyzed.

### Statistical analysis

All data are reported as the mean ± standard deviation. Group mean values were compared by Student’s unpaired two-tailed t test.

### Reagents

WNT3A, Dickkopf-related protein 1 (DKK1), macrophage colony-stimulating factor (M-CSF), and soluble Receptor activator of nuclear factor kappa-B ligand (sRANKL) recombinant proteins were purchased from R&D Systems (Minneapolis, MN).

## Additional Information

**How to cite this article**: Ruiz, P. *et al. CathepsinKCre* mediated deletion of *βcatenin* results in dramatic loss of bone mass by targeting both osteoclasts and osteoblastic cells. *Sci. Rep.* **6**, 36201; doi: 10.1038/srep36201 (2016).

**Publisher’s note:** Springer Nature remains neutral with regard to jurisdictional claims in published maps and institutional affiliations.

## Supplementary Material

Supplementary Information

## References

[b1] Hartmann C. A Wnt canon orchestrating osteoblastogenesis. Trends Cell Biol. 16, 151–158 (2006). 16466918 10.1016/j.tcb.2006.01.001

[b2] Baron R. & Kneissel M. WNT signaling in bone homeostasis and disease: from human mutations to treatments. Nat. Med. 19, 179–192 (2013). 23389618 10.1038/nm.3074

[b3] Gong Y. . LDL receptor-related protein 5 (LRP5) affects bone accrual and eye development. Cell 107, 513–523 (2001). 11719191 10.1016/s0092-8674(01)00571-2

[b4] Boyden L. M. . High Bone Density Due To a Mutation in LDL-Receptor–Related Protein 5. N. Engl. J. Med. 337, 509–515 (2002). 10.1056/NEJMoa013444 12015390

[b5] Zylstra C. R. . Gene targeting approaches in mice: assessing the roles of LRP5 and LRP6 in osteoblasts. J. Musculoskelet. Neuronal Interact. 8, 291–293 (2008). 19147944

[b6] Hu H. . Sequential roles of Hedgehog and Wnt signaling in osteoblast development. Development 132, 49–60 (2005). 15576404 10.1242/dev.01564

[b7] Day T. F., Guo X., Garrett-Beal L. & Yang Y. Wnt/β-catenin signaling in mesenchymal progenitors controls osteoblast and chondrocyte differentiation during vertebrate skeletogenesis. Dev. Cell 8, 739–750 (2005). 15866164 10.1016/j.devcel.2005.03.016

[b8] Rodda S. J. & McMahon A. P. Distinct roles for Hedgehog and canonical Wnt signaling in specification, differentiation and maintenance of osteoblast progenitors. Development 133, 3231–3244 (2006). 16854976 10.1242/dev.02480

[b9] Hill T. P., Später D., Taketo M. M., Birchmeier W. & Hartmann C. Canonical Wnt/β-catenin signaling prevents osteoblasts from differentiating into chondrocytes. Dev. Cell 8, 727–738 (2005). 15866163 10.1016/j.devcel.2005.02.013

[b10] Glass D. A. . Canonical Wnt signaling in differentiated osteoblasts controls osteoclast differentiation. Dev. Cell 8, 751–764 (2005). 15866165 10.1016/j.devcel.2005.02.017

[b11] Kramer I. . Osteocyte Wnt/beta-catenin signaling is required for normal bone homeostasis. Mol. Cell. Biol. 30, 3071–3085 (2010). 20404086 10.1128/MCB.01428-09 PMC2876685

[b12] Wang B. . Chondrocyte β-catenin signaling regulates postnatal bone remodeling through modulation of osteoclast formation in a murine model. Arthritis Rheumatol. 66, 107–120 (2014). 24431282 10.1002/art.38195 PMC3932359

[b13] Qiang Y. . Characterization of Wnt/beta-catenin signalling in osteoclasts in multiple myeloma. Br J Haematol 148, 726–738 (2010). 19961481 10.1111/j.1365-2141.2009.08009.x PMC3683858

[b14] Modarresi R., Xiang Z., Yin M. & Laurence J. WNT/beta-catenin signaling is involved in regulation of osteoclast differentiation by human immunodeficiency virus protease inhibitor ritonavir: relationship to human immunodeficiency virus-linked bone mineral loss. Am. J. Pathol. 174, 123–135 (2009). 19095956 10.2353/ajpath.2009.080484 PMC2631325

[b15] Otero K. . TREM2 and b-catenin regulate bone homeostasis by controlling the rate of osteoclastogenesis. J. Immunol. 29, 997–1003 (2012). 10.4049/jimmunol.1102836 PMC3732181 22312126

[b16] Albers J. . Canonical Wnt signaling inhibits osteoclastogenesis independent of osteoprotegerin. J. Cell Biol. 200, 537–549 (2013). 23401003 10.1083/jcb.201207142 PMC3575535

[b17] Wei W. . Biphasic and Dosage-Dependent Regulation of Osteoclastogenesis by β-Catenin. Mol. Cell. Biol. 31, 4706–4719 (2011). 21876000 10.1128/MCB.05980-11 PMC3232928

[b18] Weivoda M. M. . Wnt Signaling Inhibits Osteoclast Differentiation by Activating Canonical and Noncanonical cAMP/PKA Pathways. J. Bone Miner. Res. 31, 65–75 (2016). 26189772 10.1002/jbmr.2599 PMC4758681

[b19] Clausen B. E., Burkhardt C., Reith W., Renkawitz R. & Förster I. Conditional gene targeting in macrophage and granulocytes using LysMcre mice. Transgenic Res. 96, 317–330 (1999). 10.1023/a:1008942828960 10621974

[b20] Chiu W. S. M. . Transgenic mice that express Cre recombinase in osteoclasts. Genesis 39, 178–185 (2004). 15282744 10.1002/gene.20041

[b21] Nakamura T. . Estrogen Prevents Bone Loss via Estrogen Receptor α and Induction of Fas Ligand in Osteoclasts. Cell 130, 811–823 (2007). 17803905 10.1016/j.cell.2007.07.025

[b22] Okamoto M. . Conditional deletion of Bmpr1a in differentiated osteoclasts increases osteoblastic bone formation, increasing volume of remodeling bone in mice. J. Bone Miner. Res. 26, 2511–2522 (2011). 21786321 10.1002/jbmr.477

[b23] Brault V. . Inactivation of the beta-catenin gene by Wnt1-Cre-mediated deletion results in dramatic brain malformation and failure of craniofacial development. Development 128, 1253–1264 (2001). 11262227 10.1242/dev.128.8.1253

[b24] Holmen S. L. . Essential role of β-catenin in postnatal bone acquisition. J. Biol. Chem. 280, 21162–21168 (2005). 15802266 10.1074/jbc.M501900200

[b25] Huelsken J. . Requirement for beta-catenin in anterior-posterior axis formation in mice. J. Cell Biol. 148, 567–578 (2000). 10662781 10.1083/jcb.148.3.567 PMC2174807

[b26] Chen I. & Long F. β-catenin Promotes Bone Formation And Suppresses Bone Resorption in Postnatal Growing Mice. J. Bone Miner. Res. 28(5), 1160–1169 (2013). 23188722 10.1002/jbmr.1834 PMC3631304

[b27] Dwek J. R. The periosteum: what is it, where is it, and what mimics it in its absence? Skeletal Radiol. 39, 319–323 (2010). 20049593 10.1007/s00256-009-0849-9 PMC2826636

[b28] Roberts S. J., Gastel N. V., Carmeliet G. & Luyten F. P. Uncovering the periosteum for skeletal regeneration: The stem cell that lies beneath. Bone 70, 10–18 (2015). 25193160 10.1016/j.bone.2014.08.007

[b29] O’Driscoll S. W., Saris D. B. F., Ito Y. & Fitzimmons J. S. The chondrogenic potential of periosteum decreases with age. J. Ortopaedic Res. 19, 95–103 (2001). 10.1016/S0736-0266(00)00014-0 11332626

[b30] Lutgens S. P. M., Cleutjens K. B. J. M., Daemen M. J. A. P. & Heeneman S. Cathepsin cysteine proteases in cardiovascular disease. The FASEB Journal 21, 3029–3041 (2007). 17522380 10.1096/fj.06-7924com

[b31] Gowen M. . Cathepsin K Knockout Mice Develop Osteopetrosis Due to a Deficit in Matrix Degradation but Not Demineralization. J. Bone Miner. Res. 14, 1654–1663 (1999). 10491212 10.1359/jbmr.1999.14.10.1654

[b32] Li C. Y. . Mice Lacking Cathepsin K Maintain Bone Remodeling but Develop Bone Fragility Despite High Bone Mass. J. Bone Miner. Res. 21 (2006). 10.1359/jbmr.060313 16753017

[b33] Manolagas S. C. Corticosteroids and fractures: a close encounter of the third cell kind. J. Bone Miner. Res. 15, 1001–1005 (2000). 10841168 10.1359/jbmr.2000.15.6.1001

[b34] Martin-Millan M. . The estrogen receptor-alpha in osteoclasts mediates the protective effects of estrogens on cancellous but not cortical bone. Mol. Endocrinol. 24, 323–334 (2010). 20053716 10.1210/me.2009-0354 PMC2817608

[b35] Almeida M., Han L., Bellido T., Manolagas S. C. & Kousteni S. Wnt proteins prevent apoptosis of both uncommitted osteoblast progenitors and differentiated osteoblast by a-catenin-dependent and -independent signaling cascades involving Src/ERK and phosphatidylinositol 3-kinase/AKT. J. Biol. Chem. 280, 41342–41351 (2005). 16251184 10.1074/jbc.M502168200

[b36] Zhang Z. . Secreted frizzled related protein 2 protects cells from apoptosis by blocking the effect of canonical Wnt3a. J Mol Cell Cardiol 46, 370–377 (2009). 19109969 10.1016/j.yjmcc.2008.11.016 PMC2710029

[b37] Zimmerman Z. F., Kulikauskas R. M., Bomsztyk K., Moon R. T. & Chien A. J. Activation of Wnt/β-catenin signaling increases apoptosis in melanoma cells treated with trail. PLoS One 8, e69593 (2013). 23869245 10.1371/journal.pone.0069593 PMC3711908

[b38] Song L. . Loss of Wnt/b-catenin signaling causes cell fate shift of preosteoblasts from osteoblasts to adipocytes. Changes 29, 997–1003 (2012). 10.1002/jbmr.1694 PMC3474875 22729939

[b39] Qing H. . Demonstration of Osteocytic Perilacunar/Canalicular Remodeling in Mice during Lactation. J Bone Miner. Res. 27, 1018–1029 (2012). 22308018 10.1002/jbmr.1567 PMC3770147

[b40] Sánchez-Duffhues G., Hiepen C. & Knaus P. Bone morphogenetic protein signaling in bone homeostasis. Bone 80, 43–59 (2015). 26051467 10.1016/j.bone.2015.05.025

[b41] Chen Y. . B-Catenin Signaling Pathway Is Crucial for Bone Morphogenetic Protein 2 to Induce New Bone Formation. Journal of Biological Chemistry 282, 526–533 (2007). 17085452 10.1074/jbc.M602700200

[b42] Kamiya N. . Disruption of BMP signaling in osteoblasts through type IA receptor (BMPRIA) increases bone mass. J. Bone Miner. Res. 23, 2007–2017 (2008). 18684091 10.1359/JBMR.080809 PMC2686924

[b43] Tang N. . BMP-9-induced osteogenic differentiation of mesenchymal progenitors requires functional canonical Wnt/b-catenin signalling. J. Cell. Mol. Med. 13, 2448–2464 (2009). 19175684 10.1111/j.1582-4934.2008.00569.x PMC4940786

[b44] Itoh K. . Bone Morphogenetic Protein 2 Stimulates Osteoclast Differentiation and Survival Supported by Receptor Activator of Nuclear Factor-kB Ligand. Endocrinology 142, 3656–3662 (2016). 10.1210/endo.142.8.8300 11459815

[b45] Abe E. . Essential Requirement of BMPs-2/4 for Both Osteoblast and Osteoclast Formation in Murine Bone Marrow Cultures from Adult Mice: Antagonism by Noggin. J. Bone Miner. Res. 15, 663–673 (2000). 10780858 10.1359/jbmr.2000.15.4.663

[b46] Zhang R. . Wnt/β-catenin signaling activates bone morphogenetic protein 2 expression in osteoblasts. Bone 52, 145–156 (2013). 23032104 10.1016/j.bone.2012.09.029 PMC3712130

[b47] Yang W. . Ptpn11 deletion in a novel progenitor causes metachondromatosis by inducing hedgehog signalling. Nature 499, 491–495 (2013). 23863940 10.1038/nature12396 PMC4148013

[b48] Livak K. J. & Schmittgen T. D. Analysis of relative gene expression data using real-time quantitative PCR and the 2(−ΔΔ C(T)) Method. Methods 25, 402–408 (2001). 11846609 10.1006/meth.2001.1262

