# Supplementary material for: CathepsinKCre mediated deletion of βcatenin results in dramatic loss of bone mass by targeting both osteoclasts and osteoblastic cells
Source: Sci Rep. 2016 Nov 2;6:36201. doi: 10.1038/srep36201 (PMC5090355; doi:10.1038/srep36201)
Supplement: Supplementary Information [file srep36201-s1.pdf]

CathepsinKCre mediated deletion o*f βcatenin* results in dramatic loss of bone mass by targeting both osteoclasts and osteoblastic cells

Paula Ruiz[1], Marta Martin-Millan[1,2], MC Gonzalez-Martin[3], Maria Almeida[4], Jesús González Macias[1,2†] & Maria A. Ros[3,5*]

(1) Instituto de Investigación Marqués de Valdecilla, IDIVAL

(2) Department of Internal Medicine, HUMV, Hospital Universitario Marqués de Valdecilla, Avenida de Valdecilla s/n, 39008 Santander, Cantabria, Spain

(3) Instituto de Biomedicina y Biotecnología de Cantabria, IBBTEC (CSIC-Universidad de Cantabria). 39011 Santander. Spain.

(4) University of Arkansas

(5) Departamento de Anatomía y Biología Celular. Facultad de Medicina. Universidad de Cantabria. 39011 Santander.

Correspondence to: rosm@unican.es; mirgmj@humv.es

Supplementary figure 1

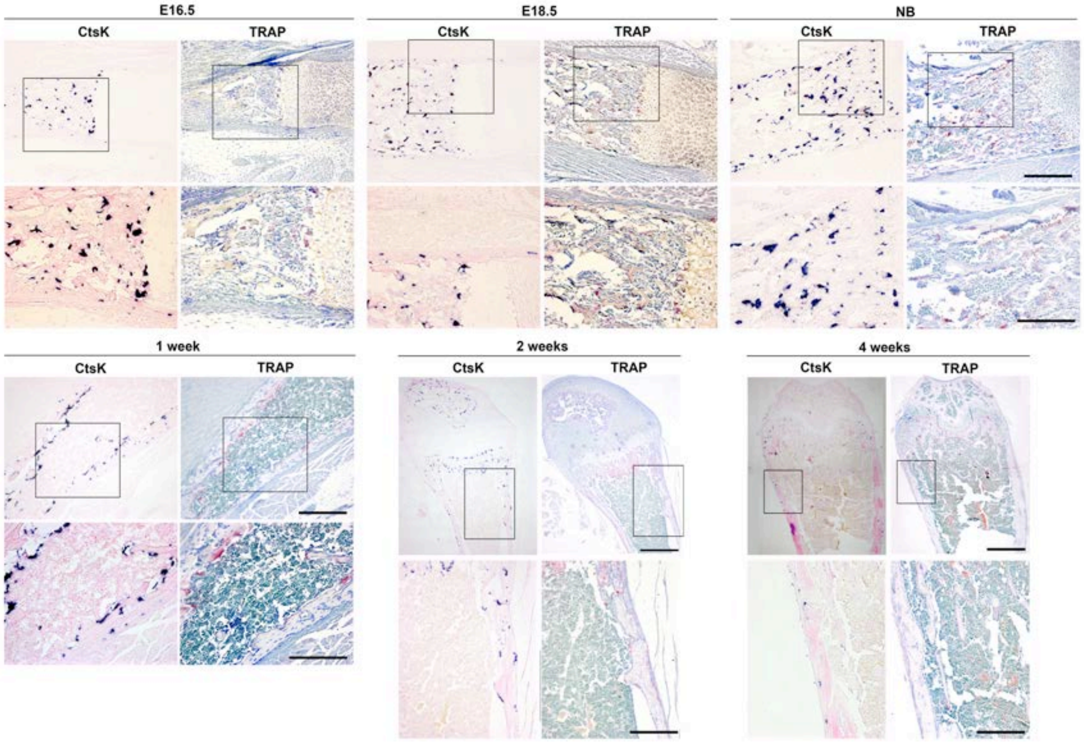

Comparison of the pattern of expression of *CtsK* and TRAP staining during bone prenatal and postnatal development

In situ hybridization for *CtsK* and TRAP staining in consecutive histological sections of the proximal femur of wild type animals for comparison. The squared region is amplified in the bottom row. Stages indicated at the top.
